# miR-21-KO Alleviates Alveolar Structural Remodeling and Inflammatory Signaling in Acute Lung Injury

**DOI:** 10.3390/ijms21030822

**Published:** 2020-01-27

**Authors:** Johanna Christine Jansing, Jan Fiedler, Andreas Pich, Janika Viereck, Thomas Thum, Christian Mühlfeld, Christina Brandenberger

**Affiliations:** 1 Institute of Functional and Applied Anatomy, Hannover Medical School, 30625 Hannover, Germany; 2 Biomedical Research in Endstage and Obstructive Lung Disease Hannover (BREATH), Member of the German Center for Lung Research (DZL), 30625 Hannover, Germany; 3 Institute of Molecular and Translational Therapeutic Strategies, Hannover Medical School, 30625 Hannover, Germany; 4 REBIRTH Center for Translational Regenerative Medicine, 30625 Hannover, Germany; 5 Institute of Toxicology, Hannover Medical School, 30625 Hannover, Germany

**Keywords:** acute lung injury, microRNA-21, alveolar micromechanics, structural remodeling, inflammatory signaling

## Abstract

Acute lung injury (ALI) is characterized by enhanced permeability of the air–blood barrier, pulmonary edema, and hypoxemia. MicroRNA-21 (miR-21) was shown to be involved in pulmonary remodeling and the pathology of ALI, and we hypothesized that miR-21 knock-out (KO) reduces injury and remodeling in ALI. ALI was induced in miR-21 KO and C57BL/6N (wildtype, WT) mice by an intranasal administration of 75 µg lipopolysaccharide (LPS) in saline (*n* = 10 per group). The control mice received saline alone (*n* = 7 per group). After 24 h, lung function was measured. The lungs were then excised for proteomics, cytokine, and stereological analysis to address inflammatory signaling and structural damage. LPS exposure induced ALI in both strains, however, only WT mice showed increased tissue resistance and septal thickening upon LPS treatment. Septal alterations due to LPS exposure in WT mice consisted of an increase in extracellular matrix (ECM), including collagen fibrils, elastic fibers, and amorphous ECM. Proteomics analysis revealed that the inflammatory response was dampened in miR-21 KO mice with reduced platelet and neutrophil activation compared with WT mice. The WT mice showed more functional and structural changes and inflammatory signaling in ALI than miR-21 KO mice, confirming the hypothesis that miR-21 KO reduces the development of pathological changes in ALI.

## 1. Introduction

Acute lung injury (ALI) or acute respiratory distress syndrome (ARDS) results from severe alveolar injury with increased permeability of the alveolar–capillary barrier, which is most often caused by pneumonia or sepsis. The pathophysiology of the disease is characterized by a strong pulmonary inflammation with a diffuse alveolar damage and edema formation that leads to decreased pulmonary compliance, hypoxemia, and respiratory failure (reviewed in [1]). The mortality rate is still relatively high, and recovery is often associated with an increased risk of developing pulmonary fibrosis [2,3]. As of now, however, treatment options are limited to supportive care with mechanical ventilation, since pharmaceutical treatment options are still lacking [4].

MicroRNAs are small non-coding RNA sequences that regulate the gene expression of cellular processes, such as cell proliferation, differentiation, cell metabolism, or apoptosis, by forming complementary sequences to mRNAs and thereby inhibiting their translation (reviewed in [5,6]). MicroRNA-21 (miR-21) has been described to have pro-inflammatory, proliferative, and fibrotic activities in tumor development or fibrosis in a variety of different tissues and organs [7,8,9]. It was shown to be transcriptionally regulated via AP-1, STAT3, or NFκB signaling and interacting with a series of pathways such as PTEN/AKT, SMAD/TGFβ, PDCD4, and others (reviewed in [6,10]). In the lung, miR-21 was found to be upregulated and involved in the development of ALI and pulmonary fibrosis [11,12]. Patients with ARDS showed increased levels of miR-21 in the blood serum [13] and miR-21 was upregulated in different murine models of ALI [13,14,15,16,17]. This was also the case in patients with idiopathic pulmonary fibrosis (IPF) and in murine models of bleomycin-induced lung injury and fibrosis [12,18,19]. Previous studies have shown that the inhibition of miR-21 signaling improved lung function and oxygenation and reduced the formation of edema in ventilation-induced lung injury (VILI) or lipopolysaccharide- (LPS) induced ALI [13,17]. Other studies, however, also indicated that miR-21 could have beneficial effects on the development of ALI [20,21]. Together these studies provide evidence that miR-21 is involved in the development of ALI, however, its molecular role and the underlying mechanisms are still poorly understood.

While these previous studies have addressed the impact of miR-21 signaling in ALI by using synthetic overexpression (agomiR-21) or inhibition (antagomiR-21), in the current study miR-21 knock-out (KO) mice were used, with the advantage of a specific and permanent loss of miR-21 in the adult system. The impact of miR-21 was addressed in the acute phase of LPS-induced ALI, and we hypothesized that miR-21 KO ameliorates pulmonary inflammation and tissue damage.

## 2. Results

ALI was induced in male C57BL/6N wild-type (WT) mice and miR-21 knock-out (KO) mice by an intranasal application of 75 µg LPS per mouse (*n* = 10 per strain). Control mice received saline (*n* = 7 per strain). Twenty-four hours later, lung function was measured, mice were sacrificed, and lung injury, remodeling, and inflammation were analyzed. The MiR-21/snoRNA202 ratio was analyzed to measure relative miR-21 expression in the lung. In the KO mice, miR-21 expression was below assay sensitivity. In the WT controls, the ratio was 0.886/0.160 (mean/SD) and significantly (*p* < 0.05) increased with LPS exposure in WT mice (1.571/0.624).

### 2.1. Lung Function

**Figure 1:**
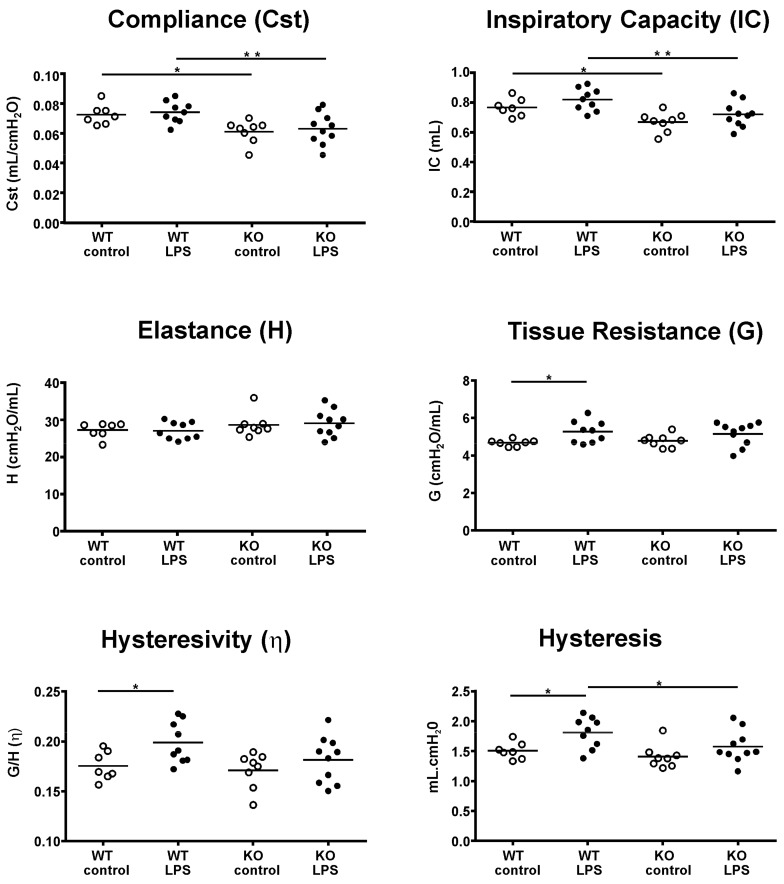
Lung function analysis. Pulmonary function and micromechanics were assessed at a positive end-expiratory pressure (PEEP) value of 3 cmH_2_O with a mouse FlexiVent (SCIREQ) ventilator in wild-type (WT) and knock-out (KO) mice with and without acute lung injury (ALI). Each data point represents one animal; means are expressed by horizontal bars; lines indicate statistically significant differences between groups (* *p* < 0.05, ** *p* < 0.01).

The results of the lung function measurements are shown in Figure 1. The measurements revealed that static pulmonary compliance (Cst) and inspiratory capacity (IC) were significantly (*p* < 0.05) higher in WT compared with KO mice. This was equally apparent in the controls and LPS-treated mice. Significant differences with LPS exposure occurred only in WT mice, including increases in tissue resistance (G), hysteresivity (η), and hysteresis. No differences in tissue elastance (H) were measured between strains or with LPS treatment.

### 2.2. Structural Changes

**Figure 2:**
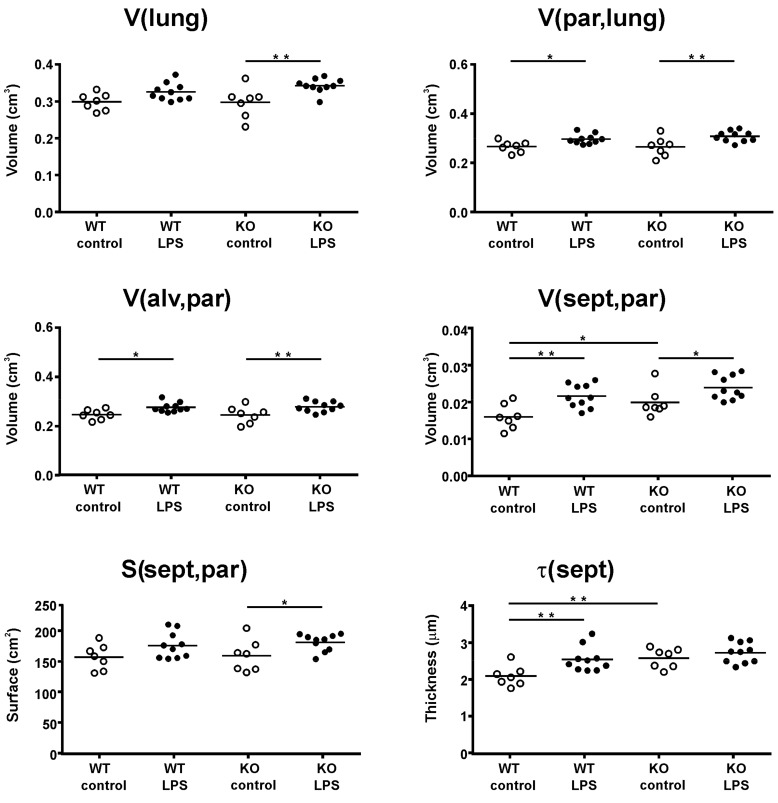
Structural alterations in lung tissue. Structural changes were assessed in the left lung lobe using stereology. The volume of the left lung lobe (V(lung)) was measured with volume displacement. The parenchymal content (V(par,lung)) and its alveolar volume (V(alv,par)) and septal volume (V(sept,par)) were estimated, as well as the septal surface area (S(sept,par)) and septal thickness (τ(sept)). Each data point represents one animal; means are expressed by horizontal bars; lines indicate statistically significant differences between groups (*p < 0.05, **p < 0.01).

**Figure 3:**
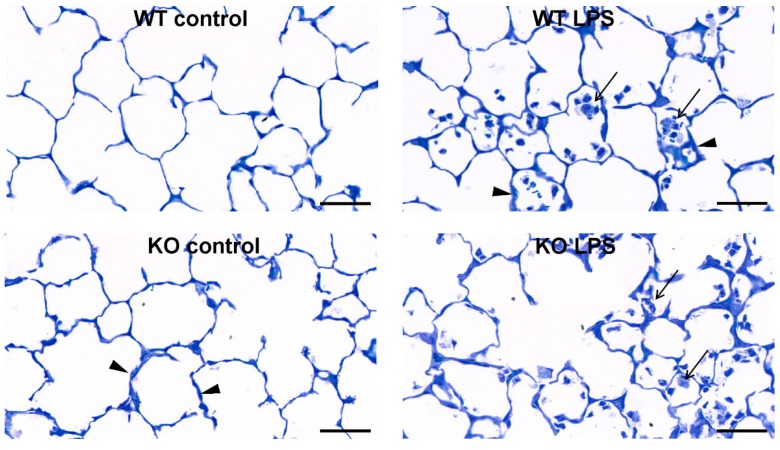
Representative light micrographs of toluidine blue stained lung parenchyma. The arrows indicate inflammatory cell infiltration, the arrow heads show septal thickening in the lung in the different experimental groups; scale bar = 50 µm.

Structural changes in the lung parenchyma were assessed by stereology (Figure 2). LPS exposure caused an increase in lung volume in KO mice (*p* = 0.002) and in WT mice (*p* = 0.054) and a significant (*p* < 0.05) increase in the parenchymal volume of both WT and KO mice. Along with the parenchymal volume, significant increases were observed in alveolar volume and septal volume with LPS exposure in both strains. In the KO mice, the effect was also accompanied by a significant (*p* = 0.04) increase in the septal surface area, which was only manifested as a trend (*p* = 0.07) in WT mice. The main difference between the strains with ALI became apparent in septal thickness (τ(sept,par)). Here, a significant (*p* = 0.004) septal thickening upon LPS exposure was measured in WT mice, but not in KO mice (*p* = 0.32). Histopathology (Figure 3) further revealed the recruitment of inflammatory cells, mostly neutrophils, into the lung tissue and alveolus.

### 2.3. Ultrastructural Septal Remodeling

**Figure 4:**
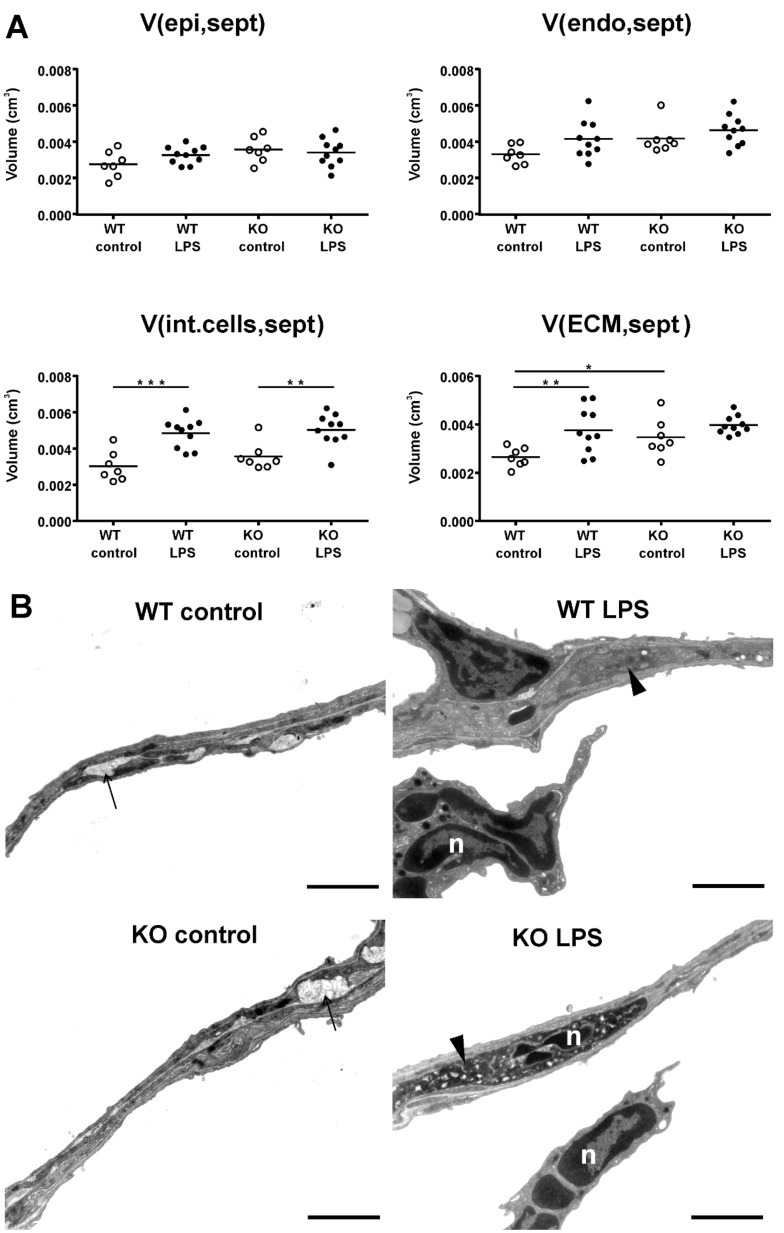
Ultrastructural changes in the pulmonary septa. The ultrastructural changes in the pulmonary septa of the left lung lobe were assessed using stereology (**A**). Analysis of the septal composition included the volume of alveolar epithelial cells (V(epi,sept)), endothelial cells (V(endo,sept)), interstitial cells (V(int.cells,sept), including fibroblasts and interstitial inflammatory cells, and ECM (V(ECM,sept) in the septa. Each data point represents one animal; means are expressed by horizontal bars; lines indicate statistically significant differences between groups (* *p* < 0.05, ** *p* < 0.01, *** *p* < 0.001). Representative transmission electron microscopy (TEM) images of the different experimental groups (**B**). The arrows show septal elastic fibers, the arrow heads point to interstitial cells, n = neutrophil, scale bar = 2 µm.

Most changes between strains with ALI were apparent in the septa; therefore, the ultrastructural septal composition was further quantified with transmission electron microscopy (TEM) and stereology. As main septal compositions, the volume of alveolar epithelial cells (V(epi,sept)), endothelial cells (V(endo,sept)), interstitial cells, including fibroblasts and recruited inflammatory cells (V(int.cell,sept)), and extracellular matrix (ECM; V(ECM,sept)) were assessed (Figure 4A). Stereological quantification revealed that no differences between strains or with ALI were apparent in alveolar epithelial and endothelial cell volume, but the volume of interstitial cells increased significantly with ALI in both WT and KO mice. The increase in interstitial cells was mostly due to inflammatory cells recruited to the septa, as also shown in representative TEM images (Figure 4B). A significant strain-related difference in septal composition with LPS exposure was measured in the volume of septal ECM. The volume of ECM was generally lower in WT compared with KO mice, but only in WT mice was a significant (p < 0.01) increase found with LPS exposure. The changes in ECM composition were therefore sub-divided into collagen fibrils (V(collagen,sept)), elastic fibers (V(elastin,sept)) and other amorphous ECM (V(amorphous ECM,sept)), as shown in Table 1. The KO mice differed from the WT mice by an increased content of amorphous ECM in the septa. In WT mice, all three ECM compartments were significantly increased with LPS exposure, but no changes with LPS exposure were found in KO mice.

### 2.4. Pulmonary Cytokine Expression

Inflammatory cytokine expression was measured in the lung tissue with a multiplex bead array. The levels of inflammatory cytokines (IFNγ, TNFα, IL-6, IL-1β, CXCL1, CCL2, and CCL5) were significantly increased with LPS exposure in both strains (Table 2). No differences were detected between strains in control mice and all cytokines, with the exception of CCL5, were equally induced in WT and KO mice after LPS exposure. CCL5, however, was 80% higher in WT compared with KO mice (*p* < 0.001).

### 2.5. Proteomics Analysis

**Figure 5:**
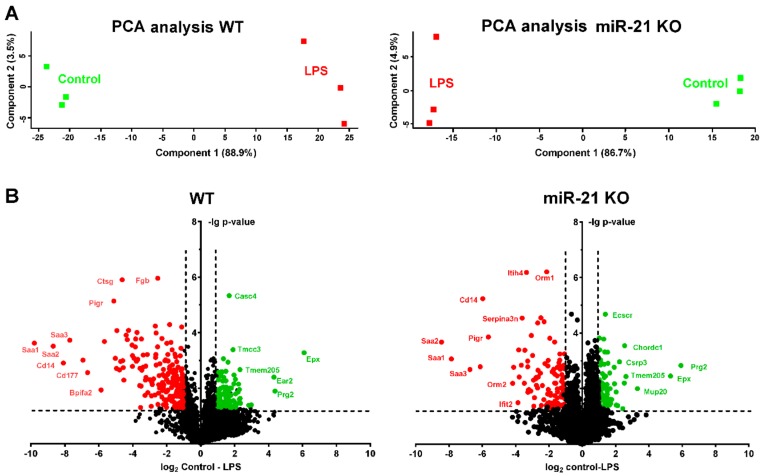
Proteome analyses of mice challenged with LPS. The total protein was isolated from the right lung lobes (n = 3 per experimental group) and proteome analyses were performed as described in the methods section. Principal component analyses of the regulated proteins showing separated groups of the replicate analyses (**A**). The volcano plots display the up- and down-regulated proteins (**B**). X-axes give the ratios of the mean protein intensities as log_2_-values. Y-axes show the –lg of the p-values. The proteins upregulated in the LPS-treated samples are shown in red, and proteins downregulated in the LPS samples are shown in green. Some selected proteins are indicated by the gene name.

A proteome analysis was conducted in WT and KO mice with LPS or control treatment (n = 3 per experimental group). The extracted proteins were digested with trypsin and analyzed with LC-MS, and proteins were identified and quantified. Overall, 4259 protein groups were identified and 3876 could be quantified in all replicates. Some missing values were imputated. Relative changes in WT mice with LPS are shown in Supplemental File 1 and changes in KO mice with LPS are shown in Supplemental File 2. With LPS exposure, 193 protein groups were significantly upregulated and 93 were significantly downregulated in samples of WT mice, while 118 protein groups were significantly upregulated and 74 were significantly downregulated in miR-21 KO mice. Principal component analysis and volcano plots of the data are shown in Figure 5. Of the significantly regulated protein groups, 70 were commonly regulated with LPS exposure in both WT and KO mice, while 212 were only regulated in WT and 121 in KO mice. A string database analysis (string-db.org) was performed for a potential pathway analysis, and the reactome pathways are shown in Table 3. The most significant reactome pathways in WT mice upon LPS exposure were “innate immune system”, “neutrophil degranulation”, and “platelet degranulation/activation”. These were also present in the miR-21 KO mice but were much more attenuated. Some signaling molecules associated with neutrophil and platelet activation, ECM remodeling or coagulation cascade (e.g., EGFR, CD44, CD63, CD177, CTSG, MMP8, MMP9, ELA2, MPO, pro-thrombin, fibrinogen, or fibronectin) were only found to be significantly upregulated in WT, but not in miR-21 KO mice with ALI. Protein groups related to “platelet degranulation”, “regulation of complement cascade”, “immune system”, and “neutrophil degranulation” were already attenuated in KO compared with WT mice without LPS exposure. Other protein groups related to “activation of DNA fragmentation”, “formation of senescence-associated heterochromatin foci“, or “apoptosis”, however, were found to be more prominent in untreated KO mice. Relative changes in KO vs WT control mice are shown in Supplemental File 3.

## 3. Discussion

MiR-21 was shown to be involved in the acute inflammatory as well as in the chronic fibrotic phase of ALI [13,19]. Herein, we addressed the impact of miR-21 on early inflammatory response and early tissue damage in ALI by comparing the degree of ALI in miR-21 KO vs WT mice. As hypothesized, the genetic loss of miR-21 reduced structural remodeling, inflammatory signaling, and lung function decline in ALI.

Previous studies have addressed the impact of miR-21 signaling in ALI by using ago- or antagomiRs to regulate miR-21 signaling, however, with controversial results [13,17,20,21]. Vaporidi and colleagues showed that mice pretreated with anti-miR-21 before the induction of VILI had better oxygenation and lower BALF protein concentrations in comparison to mice that were not pretreated with anti-miR-21, suggesting that the downregulation of miR-21 ameliorated the development of VILI [17]. Qi and colleagues also showed that miR-21 supported the inhibition of ENaC-γ, an epithelial sodium channel that is essential for the removal of edematous fluid in ALI, through PTEN/AKT signaling, and downregulation of miR-21 reversed this effect [13]. However, other studies suggested a beneficial effect of miR-21, mediated either by its anti-apoptotic potential in ischemia/reperfusion induced lung injury or by inhibiting LPS-mediated NFκB signaling in rats with ALI [20,21]. The reasons for the different study outcomes could be related to the particular injury models as well as the time points and the read-out parameters, but also to the specificity of miR-21 targeting. In comparison to these previous studies, the advantage of the genetic knock-out of miR-21 is the full and homogeneous deletion of miR-21 and the lack of side effects due to the potential reaction of the anatogonists with unspecific targets. In line with the first two studies, our results provide evidence that inflammatory signaling, structural remodeling and impaired lung function in ALI was improved by the deletion of miR-21.

The inflammatory signaling response in ALI with miR-21 KO was addressed with proteomics analysis and cytokine ELISA in lung tissue. The results show that miR-21 KO significantly dampened the inflammatory response in the lung. The reactome pathway analysis showed “innate immune system”, “neutrophil degranulation”, and “platelet degranulation/activation” to be the most prominent response clusters. These were also present in the miR-21 KO mice, but much more attenuated, and some signaling molecules associated with neutrophil and platelet activation, ECM remodeling or coagulation cascade were only found to be significantly upregulated in WT but not in miR-21 KO mice with ALI. The classic LPS-mediated TLR4/MyD88/NFκB pathway that regulates TNFα, IL-6 or CCL2 expression was, however, not significantly affected by miR-21 KO. Among the cytokines measured in the lung tissue, CCL5 was the only one which was downregulated by miR-21 KO. CCL5 is one of the main platelet-derived chemokines involved in acute inflammatory response and neutrophil activation [22,23,24]. Previous studies have shown that platelet depletion or treatment with CCL5 antibodies reduced neutrophil recruitment and permeability in LPS-induced ALI [25] and that blocking platelet-neutrophil aggregation resulted in a reversal of acid-induced ALI [26]. Hence, these studies provide evidence that platelet-mediated neutrophil activation significantly contributes to the development of ALI, and our findings suggest that this activation is reduced in miR-21 KO mice. Previous studies have suggested that miR-21 has an impact on platelet activation [27,28]. Barwari and colleagues investigated the effect of miR-21 on platelets and fibrinogenic response and showed that platelet counts and response were lowered in miR-21 KO mice, while the pharmacological inhibition of miR-21 did not affect platelet numbers but significantly decreased the release of platelet granules [27]. We did not assess platelet counts in our study, but the previous findings still support our observations that miR-21 KO reduces the platelet activation cascade, contributing to the fibrinogenic response and neutrophil activation in ALI.

Structural changes with ALI included an increase in parenchymal lung volume in both strains (WT and KO), which was associated with an increase in alveolar volume and septal volume. An increase in septal thickness with LPS treatment was only found in WT mice. These changes fit with our observations in lung function measurements, where an increase in tissue resistance (G) and hysteresis was detected. G is an inverse measure of compliance in the parenchymal lung tissue and its increase in ALI, related to septal edema formation, has been shown previously [29]. Hysteresis might increase due to changes in tissue resistance and elastance or due to changes in surfactant composition [30,31]. We further analyzed the septal composition on the ultrastructural level to characterize changes in septal tissue composition, including cells and ECM. In both strains, we detected an increase in inflammatory cells—mostly neutrophils—that were recruited to the site of injury. However, the WT mice also showed an increase in ECM volume, consisting of increased volumes of collagen fibrils, elastic fibers, and amorphous ECM, which was not present in the KO mice. These findings suggest an increase in inter-septal fluid as well as early tissue remodeling in ALI in WT, but not in KO mice, with an impact on pulmonary micromechanics. Studies addressing miR-21 in lung tissue remodeling and fibrosis have also shown that treatment with anti-miR-21 or a pharmacological inhibitor of miR-21 reduced the development of fibrosis by attenuating TGF-β signaling [12,32]. TGF-β has been reported to be modulated by miR-21 and is related to tissue remodeling and the development of fibrosis [27,33,34]. In this study, we did not detect differences in TGF-β signaling between WT and KO mice with ALI. However, it has to be considered that 24 h is a very early time point to address tissue remodeling, and the development of fibrosis usually occurs several days after induction of injury. Hence, our study provides insight into early septal remodeling with impact on lung function in ALI that is improved by deletion of miR-21.

Besides attenuating the development of ALI, miR-21 KO also caused some general changes in lung structure and function. For example, lung compliance and inspiratory capacity was reduced in miR-21 KO compared with WT mice. The lung volume was, however, approximately the same in both strains. The major differences on the structural level between the two strains were a higher septal volume and thickness in the KO mice with an increased content of amorphous ECM. It seems likely that these structural changes affected lung compliance and inspiratory capacity in KO mice; however, other parameters, such as tissue resistance or tissue elastance, that are usually affected by changes in septal composition were the same in both strains. It is therefore likely that other mechanisms, which were not covered by our analysis, were responsible for the differences between the two strains in pulmonary micromechanics. Furthermore, it is not clear which regulatory processes led to structural changes in the miR-21 KO mice. Interestingly though, any differences in pulmonary micromechanics and lung structure between WT and KO mice disappeared with progressing age (18 month old mice, Supplementary Figures S1 and S2), so it seems that there exists a compensatory mechanism for miR-21 signaling in miR-21 KO mice. This might also be in line with other reports that showed decent effects of antagomiR-21 treatment but not of genetic miR-21 deletion in cardiac tissue remodeling [27], suggesting some adaptive response to miR-21 KO.

In summary, within this project, it was shown that miR-21 contributes to inflammatory signaling and septal remodeling in ALI. In particular, we could show that miR-21 KO reduced platelet and neutrophil degranulation and septal ECM remodeling in ALI. MiR-21 KO improved lung function and dampened the inflammation in ALI, and the recent data indicate that miR-21 could be a potential therapeutic non-coding RNA target in the treatment of ALI.

## 4. Material and Methods

### 4.1. Animal Model

Male C57BL/6N mice (WT) and miR-21 knock-out mice (KO; B6N-Mir21^tm1Engl^) were bread and maintained at the local animal facility at Hannover Medical School. The KO mice were originally provided by Prof. Engelhardt, TU Munich, Germany, and the strain has been described previously in the literature by Chau et al. [35]. The mice were housed in standard cages in groups of 1–5 and had access to food and water ad libitum. At the age of 3 months, ALI was induced by intranasal administration of 75 µg LPS in 30 µL physiological saline solution (*n* = 10 per group). The control mice received equal volume of saline solution (*n* = 7 per group). The animals were sacrificed 24 h after the induction of ALI, as described previously [29]. All animal procedures were approved by the Lower Saxony State Office for Consumer Protection and Food Safety (LAVES; Authorization number: 33.9-42502-04-14/1623, Approval Date: 26/09/2014) in accordance with the German law for animal protection and with the European Directive, 2010/63/EU.

### 4.2. Lung Function Analysis 

Lung function analysis was performed with a FlexiVent rodent ventilator FX1 for mice (SCIREQ) as described previously [29]. In brief, mice were tracheotomized under deep anesthesia and mechanically ventilated with a frequency of 100 breaths/min and a tidal volume of 10 mL/kg body weight. Pulmonary function was assessed by ventilation perturbations at a positive end-expiratory pressure (PEEP) value of 3 cmH_2_O to estimate tissue elastance (H), tissue resistance (G), and hysteresivity (η) with the constant phase model to the impedance spectra. The inspiratory capacity (IC) was assessed by derecruitability maneuvers, static compliance (Cst), and hysteresis by recording quasi-static pressure-volume loops. The lung function measurement was only considered as valid if the heart was beating during measurements. 

After lung function measurements, animals were killed by exsanguination and lungs were excised. The left lung lobe was chemically fixed via intra-tracheal instillation at a pressure of 20 cmH_2_O with a fixative mixture of 1.5% paraformaldehyde and 1.5% glutaraldehyde in 0.15 M HEPES buffer for histopathology and stereological analysis. The right lung lobes were separated and immediately snap-frozen in liquid nitrogen for later processing for cytokine and proteomics analysis. 

### 4.3. Histopathology and Stereology

The left lung lobes were stored for at least 24 h in the fixative solution, followed by lung volume measurement via volume displacement and systematic uniform random subsampling (SURS) as described previously [29,36]. In brief, lung tissue was sliced in 2 mm sections and every other section, with a random starting point, was processed and embedded in glycol methacrylate (Technovit 7100 resin, Kulzer GmbH) for light microscopic analysis. The remaining lung sections were further subsampled into tissue blocks of approximately 1 mm^3^ size and embedded in epoxy resin (SERVA Electrophoresis GmbH) for electron microscopic and ultrastructural analysis. The embedding procedures were performed as described previously [29].

Stereological analyses were used to quantify the pulmonary structural characteristics and were performed in accordance with the ATS/ERS guidelines [37]. The following parameters were determined by stereology and bright-field light microscopy: volumes of parenchyma, septa, and alveoli, the surface of the alveoli, and the septal thickness as previously described in detail [29]. For the analysis, the samples embedded in glycol methacrylate were cut into 1.5 µm thick sections and stained with toluidine blue. The histological slides were then digitalized with a histological slide scanner (AxioScan.Z1, Zeiss) at a 20× magnification. The images for stereological analysis were obtained by SURS using the newCast acquisition software (Visiopharm). For parenchymal volume estimation, 50 images were sampled at a 5× magnification and analyzed with the SETPanizer software [38] with a test system of 36 points. For the other parameters, 60 images were sampled at a magnification of 20×. The images were analyzed with the STEPanizer software with a test system of 2 lines and a test system of 49 points to count alveolar airspace volume, septal volume, septal surface area, and septal thickness, as described in Kling et al. 2017 [29].

The ultrastructural analysis of the air–blood barrier was done by means of stereology and TEM assessing the following parameters: the volume of epithelial, endothelial, and interstitial cells, ECM, elastic fibers, and capillary lumen. Of the epon embedded tissue blocks, three per lung were randomly chosen, cut in ultrathin sections (60–80 nm thick), mounted on copper grids and stained with uranyl acetate and lead citrate. TEM images were acquired with a Morgagni 268 microscope (FEI) by SURS. Approximately 60 images of the air–blood barrier were recorded per tissue block at a magnification of 18,000x. The images were analyzed with the newCast software (Visiopharm, Hoersholm, Denmark) and a test system with 5 lines.

### 4.4. Gene Expression Analysis

Total RNA isolation was performed on the accessory lung lobe of the right lung with the NucleoSpin miRNA Kit (Macherey-Nagel) according to the manufacturer’s protocol. To analyze miRNA expression, two-step RT-PCR primer sets from Applied Biosystems were used according to the manufacturer’s protocol. SnoRNA-202 served as a housekeeping control. The quantification of miRNA was conducted using a VIIa7 Real-Time PCR cycler (Life Technologies, Waltham, MA, USA).

### 4.5. Cytokine Expression in Lung Tissue 

For cytokine concentration analysis, tissue from the caudal lung lobe of the right lung was mixed with 10 ml/g cOmplete^™^ (Sigma–Aldrich, Taufkirchen, Germany) in PBS and was subsequently homogenized (Tissue Lyser, Qiagen, Germantown, MD, USA). Total protein concentration of the supernatant of the lung homogenate was examined with the Pierce^TM^ BCA Protein Assay Kit (Thermo Fisher Scientific, Waltham, MA, USA). The cytokine concentration was then analyzed with the LEGENDplex bead array ELISA (# 740621, BioLegend, San Diego, CA, USA) according to the supplier’s manual and as described previously [39]. Cytokine measurements (pg/mL) were then normalized to the total protein concentration (mg/mL) to pg/mg.

### 4.6. Sample Preparation for LC-MS Analysis

Protein isolation was done with the NucleoSpin RNA/Protein Kit (Macherey-Nagel) on the cranial and middle lobes of the right lung according to the manufacturer’s protocol. The total protein concentration was measured with the Pierce BCA Protein Assay Kit (Thermo Fisher Scientific). Isolated protein suspension was then mixed with Laemmli buffer and incubated for 5 min at 95°C. Proteins were then alkylated by the addition of acrylamide up to a concentration of 2% and incubation at RT for 30 min. Afterwards, SDS-PAGE proteins were stained with Coomassie Brilliant Blue (CBB). Each lane was cut into four pieces, which were further minced into 1 mm³ gel pieces. Further sample processing was done as described [40]. Briefly, gel pieces were destained two times with 200 µL 50% and 50 mM ammonium bicarbonate (ABC) at 37°C for 30 min and were then dehydrated with 100% ACN. The solvent was removed in a vacuum centrifuge and 100 µL 10 ng/ µL sequencing grade Trypsin (Promega) in 10% ACN, 40 mM ABC were added. Gels were rehydrated in trypsin solution for 1 hour on ice and then covered with 10% ACN, 40 mM ABC. Digestion was performed over night at 37°C and was stopped by adding 100 µL of 50% ACN, 0.1% TFA. After incubation at 37°C for 1 hour, the solution was transferred into a fresh sample vial. This step was repeated twice and extracts were combined and dried in a vacuum centrifuge. Dried peptide extracts were redissolved in 30 µL 2% ACN, 0.1% TFA, with shaking at 800 rpm for 20 min. After centrifugation at 20,000× *g*, aliquots of 12.5 µL each were stored at −20 °C.

### 4.7. LC-MS Analysis

LC-MS analyses were done as described elsewhere [40]. Briefly, peptide samples were separated with a nano-flow ultrahigh pressure liquid chromatography system (RSLC, Thermo Scientific) equipped with a trapping column (3 µm C18 particle, 2 cm length, 75 µm ID, Acclaim PepMap, Thermo Scientific) and a 50 cm separation column (2 µm C18 particle, 75 µm ID, Acclaim PepMap, Thermo Scientific). Peptide mixtures were injected, enriched, and desalted on the trapping column at a flow rate of 6 µL/min with 0.1% TFA for 5 min. The trapping column was switched online with the separating column and peptides were eluted from the separating column with a multi-step binary gradient of buffer A (0.1% formic acid) and buffer B (80% ACN, 0.1% formic acid). The flow rate was 250 nL/min and the column temperature was set to 45°C. The RSLC system was coupled online via a Nano Spray Flex Ion Soure II (Thermo Scientific) to an LTQ-Orbitrap Velos mass spectrometer that was operated in data-dependent acquisition mode. Overview scans were acquired at a resolution of 60k in the orbitrap analyzer. The top 10 most intensive ions were selected for CID fragmentation in the LTQ. Active exclusion was activated so that ions fragmented once were excluded from further fragmentation for 70 s within a mass window of 10 ppm of the specific m/z value. 

The raw data were processed using Max Quant software [41] and the entries of mouse uniprot data base, including common contaminants. The proteins were identified by a false discovery rate of 0.01 on protein and peptide level and quantified by extracted ion chromatograms of all peptides. Data visualizations were done with Perseus [42] and GraphPad Prism software.

### 4.8. Statistical Analysis

Statistical analyses were conducted with the SigmaPlot software (SYSTAT Software Inc; San Jose, USA) by a two-way analysis of variance (ANOVA) followed by a pair-wise comparison with Bonferroni-test. The data that were not normally distributed were subject to a ln or square root transformation. If data normalization failed, ANOVA on Ranks followed by a Mann–Whitney U *t*-test with a Bonferroni correction was performed.

## Figures and Tables

**Table 1:** Septal extracellular matrix (ECM) composition in the left lung lobe. Septal ECM compositions were ultrastructurally quantified as either collagen fibrils (V(collagen,sept)), elastic fibers (V(elastin,sept)), or other amorphous ECM (V(amorphous ECM,sept)). The results are displayed as mean / SD. **L** = significant lipopolysaccharide (LPS) effect in the respective strain group, **S** = significant strain effect in respective exposure group; * *p* < 0.05, ** *p* < 0.01.

Group	V(collagen,sept) [mm^3^]	V(elastin,sept) [mm^3^]	V(amorphous ECM,sept) [mm^3^]
**WT control**	0.3 / 0.10	0.9 / 0.15	1.5 / 0.28
**WT LPS**	0.4 / 0.13 L**	1.3 / 0.40 L**	2.0 / 0.51 L*
**KO control**	0.4 / 0.16	1.0 / 0.29	2.0 / 0.46 S*
**KO LPS**	0.5 / 0.14	1.3 / 0.19	2.2 / 0.26

**Table 2:** Inflammatory cytokine expression in lung tissue. Cytokine expression was normalized to total tissue protein concentration (pg/mg) and is displayed as mean / SD. **L** = significant LPS effect in the respective strain group, **S** = significant strain effect in respective exposure group; * *p* < 0.05, ** *p* < 0.01, *** *p* < 0.001.

Cytokines	WT Control	WT LPS	KO Control	KO LPS
**IFN** **γ**	0.08 / 0.01	11.94 / 16.67 L*	0.08 / 0.04	10.07 / 9.81 L*
**TNF** **α**	0.52 / 0.14	45.21 / 28.05 L***	0.50 / 0.21	41.51 / 28.25 L***
**IL-6**	1.43 / 0.37	43.60 / 31.16 L**	1.19 / 0.49	32.05 / 14.97 L**
**IL-1** **β**	21.37 / 3.84	88.85 / 40.58 L***	18.94 / 5.07	75.70 / 24.49 L***
**CXCL1**	10.19 / 4.18	1239.68 / 652.86 L***	12.15 / 10.72	1241.02 / 563.06 L***
**CCL2**	1.56 / 0.43	84.29 / 34.42 L***	1.85 / 0.67	67.63 / 23.20 L***
**CCL5**	68.49 / 16.19	269.64 / 60.60 L***	60.76 / 17.08	150.07 / 40.47 L***/ S***

**Table 3:** Transcriptomics pathway analysis. Data showing reactome pathway description extracted from string database analysis (false discovery rate *p* < 0.01).

WT GO-Term	Reactome Pathway Description	Count in Gene Set	False Discovery Rate
MMU-168249	Innate Immune System	70 of 879	1.67 × 10^−32^
MMU-168256	Immune System	80 of 1523	3.30 × 10^−26^
MMU-6798695	Neutrophil degranulation	47 of 476	6.37 × 10^−25^
MMU-114608	Platelet degranulation	22 of 121	4.71 × 10^−16^
MMU-76002	Platelet activation, signaling and aggregation	26 of 242	3.03 × 10^−14^
MMU-109582	Hemostasis	34 of 489	1.26 × 10^−13^
MMU-381426	Regulation of Insulin-like Growth Factor (IGF)transport and uptake	19 of 129	1.48 ×10^−12^
MMU-977606	Regulation of Complement cascade	13 of 41	3.89 × 10^−12^
MMU-8957275	Post-translational protein phosphorylation	17 of 114	2.16 × 10^−11^
MMU-5686938	Regulation of TLR by endogenous ligand	8 of 13	3.26 × 10^−9^
MMU-140877	Formation of Fibrin Clot (Clotting Cascade)	10 of 34	4.07 × 10^−9^
MMU-1474244	Extracellular matrix organization	16 of 246	5.55 × 10^−6^
MMU-216083	Integrin cell surface interactions	9 of 68	1.36 × 10^−5^
MMU-6803157	Antimicrobial peptides	9 of 69	1.43 × 10^−5^
MMU-140875	Common Pathway of Fibrin Clot Formation	6 of 20	1.72 × 10^−5^
MMU-76009	Platelet Aggregation (Plug Formation)	6 of 29	0.0001
MMU-166665	Terminal pathway of complement	4 of 7	0.00018
MMU-140837	Intrinsic Pathway of Fibrin Clot Formation	5 of 20	0.00028
MMU-354192	Integrin alphaIIb beta3 signaling	5 of 22	0.0004
MMU-354194	GRB2: SOS provides linkage to MAPK signaling for integrins	4 of 11	0.00058
MMU-6799990	Metal sequestration by antimicrobial proteins	3 of 3	0.00073
MMU-372708	p130Cas linkage to MAPK signaling for integrins	4 of 12	0.00073
MMU-168898	Toll-like Receptor Cascades	9 of 131	0.0011
MMU-202733	Cell surface interactions at the vascular wall	8 of 103	0.0012
MMU-1566948	Elastic fiber formation	5 of 37	0.0027
MMU-1236973	Cross-presentation of particulate exogenous antigens (phagosomes)	3 of 7	0.0035
**KO GO-term**	**Reactome Pathway description**	**Count in Gene Set**	**False Discovery Rate**
MMU-6798695	Neutrophil degranulation	17 of 476	0.00024
MMU-168249	Innate Immune System	22 of 879	0.00088
MMU-76002	Platelet activation, signaling and aggregation	11 of 242	0.00094
MMU-2173782	Binding and Uptake of Ligands by Scavenger Receptors	5 of 31	0.00094
MMU-114608	Platelet degranulation	8 of 121	0.00094
MMU-2168880	Scavenging of heme from plasma	4 of 19	0.0016
MMU-168256	Immune System	27 of 1523	0.0073

## Supplementary Materials

Supplementary materials can be found at https://www.mdpi.com/1422-0067/21/3/822/s1.
