# Supplementary material for: miR-21-KO Alleviates Alveolar Structural Remodeling and Inflammatory Signaling in Acute Lung Injury
Source: Int J Mol Sci. 2020 Jan 27;21(3):822. doi: 10.3390/ijms21030822 (PMC7037600; doi:10.3390/ijms21030822)
Supplement: Supplementary file 1 [file ijms-21-00822-s001.pdf]

International Journal of
Molecular Sciences

MDPI

Supplemental Information

1. Supplemental Information on Proteome Analysis

1.1. Supplemental File 1.

Relative changes in WT mice with LPS exposure. Protein groups that are significantly and 2 fold or more induced are highlighted in dark red, and protein groups that are 2 fold or more but not significantly induced are highlighted in light red. Protein groups that are significantly and 2 fold or more reduced are highlighted in dark green, and protein groups that are 2 fold or more but not significantly reduced, are highlighted in light green.

1.2. Supplemental File 2.

Relative changes in KO mice with LPS exposure. Protein groups that are significantly reduced and 2 fold or more induced are highlighted in dark red, and protein groups that are 2 fold or more, but not significantly induced are highlighted in light red. Protein groups that are significantly and 2 fold or more reduced are highlighted in dark green, and protein groups that are 2 fold or more, but not significantly reduced, are highlighted in light green.

1.3. Supplemental File 3.

Relative changes in KO vs WT control mice are shown in Supplemental File 3. Protein groups that are significantly and 2 fold or more induced are highlighted in dark red. Protein groups that are significantly and 2 fold or more reduced are highlighted in dark green.

2. Supplemental Figures on Lung Function and Structure in 18-Month-Old WT and miR-21 KO Mice

Supplemental figures of lung structure include data that was assessed from WT and miR-21 KO control mice at different ages, including 3-month-old (young) and 18-month-old (old) mice. The data indicated that strain-related differences in lung structure and function disappears with age.

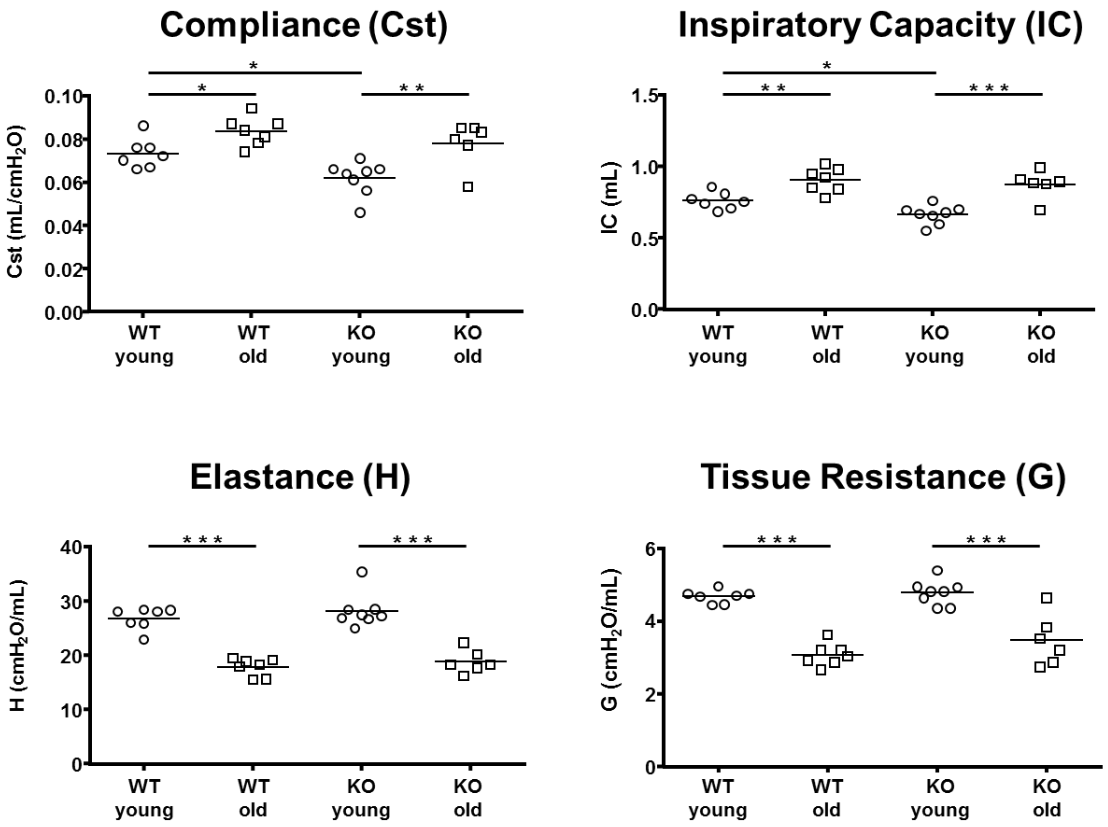

Supplemental Figure S1. Lung function analysis in young (3 months) and old (18 months) WT and miR-21 KO control mice. Each mark represents one animal. Horizontal bars represent the mean, lines show significant differences (*p < 0.05* (*), *p < 0.01* (**), *p < 0.001* (***)).

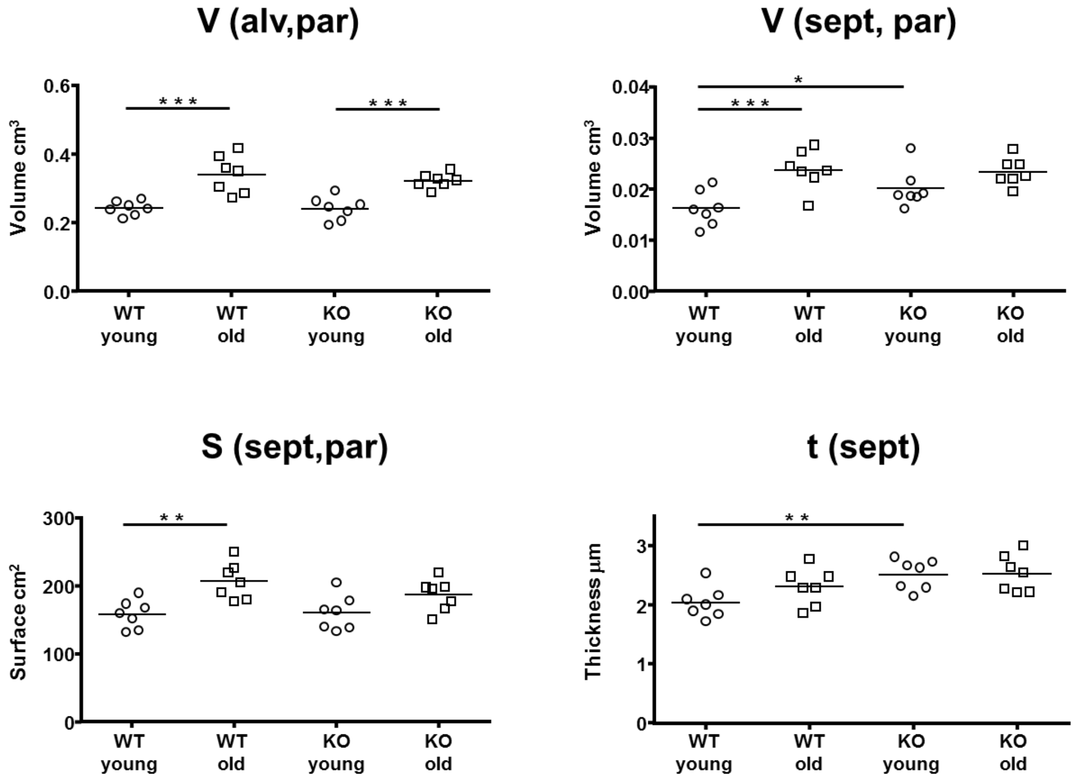

Supplemental Figure S2. Stereological analysis of lung structure in young (3 months) and old (18 months) WT and miR-21 KO control mice. Each mark represents one animal. Horizontal bars show the mean, lines indicate significant differences ($p < 0.05$ (*), $p < 0.01$ (**), $p < 0.001$ (***)).
